# Menthol versus tobacco e-liquid flavor: Impact on acute subjective effects, puff patterns, and intentions for use among Black and White menthol smokers

**DOI:** 10.1016/j.addbeh.2024.108038

**Published:** 2024-04-12

**Authors:** Eleanor L.S. Leavens, Leah M. Lambart, Gideon St.Helen, Neal L. Benowitz, Matthew S. Mayo, Kazi M. Farhad Mahmud, Michael J. Arnold, Nicole L. Nollen

**Affiliations:** a University of Kansas School of Medicine, Department of Population Health, Kansas City, KS, United States; b The University of Kansas Cancer Center, Kansas City, KS, United States; c Clinical Pharmacology Research Program, Division of Cardiology, University of California, San Francisco, CA, United States; d University of Kansas School of Medicine, Department of Biostatistics & Data Science, Kansas City, KS, United States

**Keywords:** Menthol, Electronic cigarettes, Flavor, Smoking

## Abstract

**Background:**

The proposed FDA product standard to prohibit menthol as a characterizing flavor in combustible cigarettes has the potential to significantly reduce tobacco-related health disparities. Whether a menthol e-liquid product standard would improve or hinder public health is unknown. No known research has directly examined the impact of menthol vs. tobacco flavored e-liquid use on acute e-cigarette use patterns, subjective experience, behavioral intentions, and craving and withdrawal among menthol cigarette smokers.

**Methods:**

Black (*n* = 47) and White (*n* = 4) nicotine-deprived menthol smokers with limited e-cigarette experience completed two counterbalanced in-laboratory 30-minute ad libitum vaping sessions with menthol and tobacco nicotine salt-based e-liquid in a randomized crossover pilot trial design. Questionnaires assessed reductions in craving and withdrawal and post-session subjective experience and behavioral intentions. Puff topography was measured continuously throughout each vaping session.

**Results:**

Measures of puff topography did not differ significantly by e-liquid flavor (all *p* > .40). Similarly, menthol and tobacco flavored e-cigarettes were both rated positively in terms of subjective effects and behavioral intentions (all *p* > .10) and about 40 % of participants reported a preference for the tobacco-flavored e-liquid. Finally, participants showed comparable reductions in craving (*p* = .210) and withdrawal (*p* = .671) from pre- and post-session regardless of e-liquid flavor.

**Conclusions:**

Among menthol smokers in a lab-based setting, findings suggest that menthol vs tobacco e-liquid flavor has little impact on acute changes in puff patterns, subjective experience, behavioral intentions, or craving and withdrawal.

## 1. Introduction

Menthol cigarette smoking is associated with greater initiation (Klausner, 2011) and dependence (Fagan et al., 2015) and greater difficulty quitting (D’Silva et al., 2012) compared to non-menthol cigarettes. It is estimated that from 1980 to 2018, 10.1 million people in the US initiated smoking due to the availability of menthol cigarettes (Le and Mendez, 2021). As a consequence of aggressive advertising by the tobacco industry, over 80 % of Black smokers smoke menthol cigarettes (Giovino et al., 2015), squarely placing the burden of mentholated cigarettes in minoritized communities. In 2021, the FDA proposed a product standard to prohibit menthol as a characterizing flavor in combustible cigarettes, a move that could greatly reduce the burden of tobacco use in minoritized communities (Cadham et al., 2020). Currently, FDA excludes e-cigarettes from the proposed rule. Some have argued that menthol e-cigarettes are necessary as an alternative to menthol cigarettes if the FDA is to prohibit menthol in e-cigarettes. Regulatory action involving cigarettes and e-cigarettes should strike a balance that limits uptake among youth and those naïve to nicotine while providing options for harm reduction to adult smokers. In the case of menthol in the US, regulatory action on flavored e-liquids could serve to widen tobacco-related disparities among adult smokers and concentrate the negative impacts in minoritized communities, particularly if removal of mint/menthol flavors results in reduced acceptability of e-cigarettes and leads to continued use of combusted products.

Research to date has focused on the impact of restricting menthol in combustible cigarettes and suggests substantial reductions in combustible cigarette smoking and initiation would result (Cadham et al., 2020; Levy et al., 2021). Our knowledge of the impact of restricting menthol e-cigarettes on menthol cigarette smoking behavior is limited to a few survey studies (Yang et al., 2022; White et al., 2023) and a small secondary data analysis of an e-cigarette switching trial (Nollen et al., 2022). One survey suggests that in the case of removal of menthol products, including non-combusted products like e-cigarettes, from the market, 20 % of current menthol smokers would quit all tobacco/nicotine use, 21 % would use tobacco flavored e-cigarettes, 7 % would use other tobacco products, and 52 % would switch to smoking non-menthol cigarettes, but findings did not clearly indicate whether inclusion of non-combusted products in the product standard would lead to benefit above that of a ban on combustible menthol cigarettes (Yang et al., 2022). A recent survey of Black adults who smoke found that compared to a more limited combustible ban, a comprehensive ban would result in greater intentions to quit all tobacco products and reduced intentions to switch to non-combusted products (White et al., 2023). A secondary analysis of a switching trial showed that both menthol and non-menthol e-liquid were highly acceptable and showed high rates of use and substitution (Nollen et al., 2022).

The objective of this pilot study was to investigate the acceptability and abuse liability (i.e., likelihood of product use as measured by markers of self-administration, changes in craving/withdrawal, appeal, and intentions for continued use) of menthol vs. tobacco e-cigarette e-liquid among menthol smokers. Moreover, the study aimed to ascertain potential effect sizes for these outcomes to inform future trials on the impact of menthol vs. tobacco flavored e-liquids in an acute use scenario.

## 2. Methods

### 2.1. Participants

Participants were recruited in Kansas City, MO/KS. Eligible participants were established menthol smokers who were ≥ 21 years, smoked 5–30 cigarettes per day, and were willing to use e-cigarettes. Participants were excluded if they planned to quit smoking in the next 30 days, used cessation pharmacotherapy or non-cigarette tobacco products in the past 30 days, used e-cigarettes > 5 times in their lifetime, had uncontrolled hypertension, were pregnant/breastfeeding, or were enrolled in a program to alter tobacco use.

Participants were recruited from an ongoing trial comparing nicotine pharmacokinetics of cigarettes, e-cigarettes, and heated tobacco products. Current menthol smokers were invited to participate.

### 2.2. Procedures

The study was an acute, counterbalanced, randomized crossover trial (AB, BA design) assessing acceptability and abuse liability of tobacco versus menthol e-cigarette e-liquid among current menthol smokers. Participants attended an initial visit where they were provided the study e-cigarette and both e-liquid flavors. They took the products home to practice with for ≥48 h. Participants were instructed to remain tobacco/nicotine abstinent for 12 h prior to a second study visit (expired CO ≤ 12 ppm to support abstinence), during which participants completed two 30-minute ad libitum vaping sessions, one with each e-liquid flavor. Research staff were blinded to flavor and participants were not informed which flavor they were using during each session. Vaping sessions were separated by a 90-minute washout period. The majority of participants (*n* =31) used JUUL 5 % nicotine concentration during the study, but due to regulatory action by the FDA which temporarily halted the commercial sale of JUUL during study recruitment, we used the Vuse Alto 5 % nicotine for the remaining study visits (*n* = 20). Study e-liquids had similar ingredients as reported by the manufacturer. Participants completed written informed consent. Study procedures were approved by the university’s institutional review board.

### 2.3. Measures

#### Cigarette dependence.

Baseline cigarette dependence was assessed using the Fagerström Test for Cigarette Dependence (Heatherton et al., 1991).

#### Topography.

Throughout the session, puff topography was measured via an eTop topography sensor attached to the e-cigarette.

#### Subjective effects.

Following each flavor session, participants completed measures of subjective effects. Items were rated on a visual analog scale from 0 (not at all) to 100 (extremely) and included items assessing desire, need, liking, and satisfaction. Participants completed the satisfaction and enjoyment of respiratory tract sensations subscales of the modified Cigarette Evaluation Questionnaire (mCEQ; Cappelleri et al., 2007).

#### Behavioral intentions.

Participants reported their intentions for future use of each e-liquid flavor. Each of the four items were rated on a scale from 1 (extremely unlikely) to 5 (extremely likely).

#### Cigarette craving and nicotine withdrawal changes.

Participants completed the Questionnaire of Smoking Urges-Brief (Cox et al., 2001) and Minnesota Tobacco Withdrawal Scale (Hughes and Hatsukami, 1986) prior to and immediately following each vaping session. Change scores were calculated.

#### Perceived flavoring and preference.

At the end of both vaping sessions, before being informed which flavor was used during which session, participants completed a question about their flavor preference which asked, “Of the two flavors you just used, which did you prefer?” Response options were “Flavor 1” or “Flavor 2”. Reponses were recoded to “tobacco” or “menthol”. Next, participants completed two items assessing which flavor they believed was tobacco versus menthol (“What flavor do you believe you used first/second?”). Response options were “tobacco” or “menthol”. Responses were recoded to “correct” or “incorrect” to indicate the accuracy of the participant’s guess.

### 2.4. Data analytic plan

Descriptive statistics are presented for participant characteristics and outcomes. For outcome variables, means and standard deviations are presented by sequence/treatment and combined to show overall means for the sessions. To test whether device type impacted primary outcomes, we conducted independent samples t-tests by device brand (JUUL vs. Vuse) on total inhaled volume and satisfaction ratings (mCEQ satisfaction subscale), our two primary outcomes. Tests were not significant (*p* > .20); therefore, device brands were combined for all analyses. T-tests accounting for the crossover design and repeated meaures were conducted to compare outcomes by session flavor using the methods described by Senn (2002).{Senn, 2002 #93} Analyses did not assume equal variance as this method is more robust given the relatively small sample size. QQ plots were assessed to determine if there were any major violations of non-normality for each of the variables. Only max puff volume violated the normality assumption; log transformed data are reported. Analyses were conducted using SAS version 9.4. A type I error rate of 5 % was used.

## 3. Results

### 3.1. Participant characteristics

Participants (*N* =51) were an average 54.7 (*SD* =11.1) years old and 56.9 % (n = 29) women. Ninety-two percent (*n* = 47) identified as African American/Black and 8 % (*n* = 4) as White. Participants were socioeconomically disadvantaged; most had at least a high school degree (*n* = 38; 74.6 %) but reported an annual household income of less than $25,000 (*n* = 42; 82.4 %), and 88.2 % (*n* = 45) did not own a home. Thirty-nine percent (*n* = 20) were unemployed or on disability. Participants smoked an average of 10.7 (*SD* = 5.1) cigarettes per day and reported a low level of dependence (*M* = 4.1, *SD* = 2.3, range = 0–8).

### 3.2. Outcomes (Table 1 and supplemental Table S2)

Participants took a significantly greater number of puffs when using the tobacco (*M* = 108.6, *SD* = 130.5) compared to the menthol flavored e-liquid (*M* = 85.1, *SD* = 83.8; *p* = .027). However, rhere were no statistically significant treatment effects between menthol and tobacco e-cigarette for any other measures of puff topography (all *p* > .40). There were significant period effects for some subjective effects items (i.e., pleasant, desire, want, like, pleasurable), indicating that for these items, order but not flavor significantly impacted outcomes. No significant treatment effects emerged for measures of subjective effects (all *p* > .10) or behavioral intentions (all *p* > .30). In all but one case, the second flavor was rated significantly more favorably than the first, suggesting that as naïve users acclimate to the e-cigarette, their perceptions become more favorable. Similarly, no statistically significant treatment effects were detected between the menthol and tobacco e-cigarette sessions for change in cigarette craving (*p* = .210) or nicotine withdrawal (*p* = .671).

### 3.3. Perceived flavoring and preference

Before being informed which flavored was used during each session, 39.2 % of participants reported a preference for the tobacco-flavored e-liquid compared to 60.8 % who reported a preference for menthol. Interestingly, only 60.8 % of participants were able to accurately identify which e-liquid was menthol versus tobacco flavor.

## 4. Discussion

To our knowledge, this pilot study is the first to experimentally test the acceptability and abuse liability of tobacco versus menthol flavored e-cigarette e-liquid among a sample of predominantly Black menthol smokers. Across a range of measures, smokers did not have a significant preference for either tobacco or menthol e-liquid.

Together with literature showing that flavored e-liquid was a primary catalyst of the youth e-cigarette use epidemic (King, 2020), that over 95 % of youth e-cigarette users use flavored e-liquid (Schneller et al., 2022), and that in a lab-based assessment of youth e-cigarette users, participants report more positive subjective effects and greater willingness to use a menthol/mint- versus tobacco-flavored e-cigarette (Li et al., 2022); a potential implication of the study may be that FDA authorization of menthol e-cigarettes may not be necessary. In fact, survey data suggest that a marketplace in which menthol e-cigarettes are not available may increase smokers’ intentions to quit all tobacco products (White et al., 2023). Such regulatory action could have the greatest benefit for public health by limiting youth initiation and not negatively impacting harm reduction for adult menthol smokers. Specifically, in a study of adult e-cigarette users, removal of flavors did not alter e-cigarette use patterns among adult established e-cigarette users (Yingst et al., 2021), and adult menthol smokers showed equal acceptance and uptake of non-menthol and menthol e-cigarettes in a small secondary analysis of an e-cigarette switching trial (Nollen et al., 2022). Moreover, FDA has issued the first marketing denial orders (MDOs) for menthol e-cigarette products, citing a lack of evidence that menthol e-liquid (compared to tobacco) promotes switching or reductions in cigarette smoking among adult smokers.

The current study is not without limitations. The study was limited to non-Hispanic Black and White smokers who report significant financial disadvantage. Black and White smokers comprise over 86 % of menthol smokers in the US but may not generalize to smokers of other racial/ethnic groups or those with greater financial advantage. The study was an acute assessment of two pod-based e-cigarettes and two e-liquids. Because e-liquid flavoring profiles, including menthol levels, can vary substantially, replication with additional menthol, mint, and cooling flavors in a longer-term study is needed to increase generalizability of the findings. Future studies may also investigate the impact of complete flavorant removal by examining an unflavored e-liquid. Nicotine delivery was not measured in the current study, precluding nicotine delivery comparisons by product and examination of the association between puff patterns or subjective effects and nicotine delivery. We employed a relatively short washout period. While we utilized a counterbalanced randomized crossover design to mitigate carryover effects, the study should be replicated with a longer washout period. Last, the sample size was limited. However, the largest effects size was small (d < .30), suggesting that even with a larger sample size, there would likely be little difference in the outcomes. Replication in a fully-powered trial is warranted.

The goal of regulatory action regarding menthol in e-cigarettes should be to strike a balance between protecting youth and those naïve to nicotine while leaving viable options for adult smokers who are unable to quit smoking. Among menthol smokers in a lab-based setting, findings suggest that menthol versus tobacco e-cigarette flavor has little impact on acute changes in puff patterns, subjective experience, or behavioral intentions. These findings provide initial support for prohibiting the sale of menthol e-cigarettes because they may not be a necessary alternative to menthol cigarettes among adult smokers.

## Supplementary Material

Supp table 1

## Figures and Tables

**Table 1:** Summary of overall means by e-liquid flavor and treatment effects.

Construct/Variable	Overall Mean Menthol	Overall Mean Tobacco	Treatment p-value	Effect size

	M (SD)	M (SD)		
*Puff topography*				
Total puff time, s	144.8 (134.9)	147.7 (134.2)	.895	0.020
Avg. puff duration, s	2.3 (3.7)	2.1 (2.3)	.773	0.046
Avg. flow rate, mL/s	35.0 (15.1)	34.5 (16.9)	.782	0.043
Total inhaled volume, mL	4522.9 (4148.7)	4846.2 (4849.5)	.562	0.085
Avg. puff volume	62.5 (56.7)	56.8 (38.5)	.485	0.110
Max. puff volume, mL*	4.9 (1.0)	5.0 (1.2)	.706	0.091
Avg. IPI, s	38.2 (34.8)	41.8 (39.9)	.533	0.095
Total number of puffs	85.1 (83.8)	108.6 (130.5)	.027	0.294
*Subjective effects*				
Overall experience (0–100)	49.7 (31.1)	42.2 (33.8)	.186	0.192
Pleasant (0–100)	42.5 (33.7)	41.6 (35.5)	.878	0.022
Desire (0–100)	38.6 (34.8)	34.9 (35.7)	.485	0.101
Need (0–100)	32.4 (27.9)	27.0 (27.7)	.155	0.200
Want (0–100)	34.7 (33.9)	30.3 (32.6)	.403	0.117
Like (0–100)	42.6 (34.8)	39.7 (35.6)	.619	0.071
Enjoy (0–100)	45.7 (33.8)	41.9 (37.5)	.529	0.093
Pleasurable (0–100)	43.7 (34.3)	46.9 (35.4)	.308	0.080
Satisfying (0–100)	49.1 (36.2)	48.9 (39.7)	.968	0.005
Interest in future use (0–100)	40.9 (38.7)	41.0 (40.0)	.985	0.002
Willingness for future (0–100)	42.5 (37.3)	42.8 (39.5)	.974	0.006
*mCEQ subscales*				
Satisfaction (1–21)	10.2 (5.4)	10.0 (6.1)	.819	0.031
Respiratory sensation (1–7)	3.1 (2.1)	2.9 (2.1)	.454	0.088
*Behavioral intentions*				
Try flavor again (1–5)	3.3 (1.2)	3.3 (1.5)	.983	0.000
Pay to use flavor (1–5)	2.6 (1.4)	2.8 (1.5)	.554	0.110
Purchase for personal use (1–5)	2.6 (1.4)	2.9 (1.5)	.324	0.189
Use flavor regularly (1–5)	2.6 (1.4)	2.7 (1.5)	.643	0.061
*Craving and withdrawal*				
Craving change	− 11.5 (14.4)	− 9.4 (13.5)	.210	0.250
Withdrawal change	− 2.0 (3.7)	− 2.3 (4.2)	.671	0.126

*Notes.* IPI = interpuff interval. mCEQ = Modified Cigarette Evaluation Questionnaire.

* Max puff volume values are log transformed to correct violations of the normality assumption. Effect size estimates using Cohen’s D.

## Data Availability

Data will be made available on request.
