# Supplementary material for: Menthol versus tobacco e-liquid flavor: Impact on acute subjective effects, puff patterns, and intentions for use among Black and White menthol smokers
Source: Addict Behav. Author manuscript; Available in PMC 2025 Aug 1. (PMC11221762; doi:10.1016/j.addbeh.2024.108038)
Supplement: Supp table 1 [file NIHMS2001396-supplement-Supp_table_1.docx]

| Supplemental Table 1. Summary of means and 95% CI by period and e-liquid flavor | | | | | | | | | |
| --- | --- | --- | --- | --- | --- | --- | --- | --- | --- |
|  | Menthol | | | | Tobacco | | | | Period  p-value |
|  | Period 1 | | Period 2 | | Period 1 | | Period 2 | |  |
| Construct/Variable | M (SD) | 95% CI | M (SD) | 95% CI | M (SD) | 95% CI | M (SD) | 95% CI |  |
| *Puff topography* | | | | | | | | | |
| Total puff time, s | 129.3 (102.1) | 81.4, 177.0 | 160.3 (161.2) | 90.6, 230.0 | 152.1 (119.2) | 100.6, 203.7 | 143.3 (147.2) | 74.4, 212.1 | .615 |
| Avg. puff duration, s | 1.5 (0.7) | 1.2, 1.8 | 3.04 (5.2) | 0.8, 5.3 | 2.3 (2.8) | 1.1, 3.4 | 1.9 (1.7) | 1.2, 2.7 | .376 |
| Avg. flow rate, mL/s | 33.2 (14.8) | 26.5, 39.9 | 36.8 (15.3) | 30.3, 43.3 | 35.3 (14.5) | 29.1, 41.4 | 33.7 (19.0) | 25.1, 42.4 | .568 |
| Total inhaled volume, mL | 4219.2 (3995.1) | 2349.4, 6088.9 | 4826.5 (4296.8) | 2968.4, 6684.6 | 4920.4 (4583.4) | 2938.3, 6902.4 | 4772.0 (5101.7) | 2384.4, 7159.7 | .681 |
| Avg. puff volume | 42.8 (19.4) | 33.9, 51.6 | 82.2 (77.8) | 49.3, 115.0 | 61.2 (43.3) | 42.9, 79.5 | 52.4 (33.0) | 37.4, 67.5 | .064 |
| Max. puff volume, mL* | 4.7 (0.7) | 4.4, 5.1 | 5.0 (1.2) | 4.5, 5.5 | 5.1 (1.1) | 4.6, 5.5 | 4.9 (1.2) | 4.3, 5.4 | .896 |
| Avg. IPI, s | 32.1 (25.1) | 20.7, 43.5 | 44.3 (42.3) | 26.4, 62.1 | 38.3 (34.6) | 23.7, 52.9 | 45.3 (44.6) | 25.0, 65.6 | .103 |
| Total number of puffs | 94.2 (95.1) | 50.9, 137.5 | 75.9 (70.8) | 46.0, 105.8 | 119.6 (147.6) | 57.3, 182.0 | 97.6 (110.8) | 47.2, 148.1 | .056 |
| *Subjective effects* | | | | | | | | | |
| Experience (0-100) | 50.5 (29.9) | 37.6, 63.4 | 48.8 (32.3) | 35.8, 61.9 | 39.5 (35.2) | 25.2, 53.7 | 44.8 (32.3) | 30.9, 58.8 | .740 |
| Pleasant (0-100) | 33.9 (33.0) | 19.6, 48.2 | 51.0 (34.4) | 37.2, 64.9 | 36.8 (36.8) | 22.0, 51.7 | 46.3 (34.2) | 31.5, 61.1 | .028 |
| Desire (0-100) | 29.7 (29.8) | 16.8, 42.5 | 47.5 (39.2) | 31.7, 63.4 | 30.0 (34.2) | 16.2, 43.8 | 39.7 (37.2) | 23.6, 55.7 | .013 |
| Need (0-100) | 32.3 (23.5) | 22.2, 42.5 | 32.5 (31.7) | 19.7, 45.2 | 21.7 (26.2) | 11.1, 32.3 | 32.2 (29.2) | 19.5, 44.8 | .168 |
| Want (0-100) | 29.7 (29.7) | 16.9, 42.6 | 39.7 (37.7) | 24.5, 55.0 | 23.3 (30.3) | 11.0, 35.5 | 37.2 (34.8) | 22.2, 52.3 | .029 |
| Like (0-100) | 34.3 (31.0) | 20.9, 47.7 | 50.9 (38.3) | 35.4, 66.4 | 34.7 (33.0) | 21.3, 48.0 | 44.6 (38.1) | 28.1, 61.1 | .029 |
| Enjoy (0-100) | 38.2 (30.3) | 25.1, 51.3 | 53.1 (36.9) | 38.2, 68.0 | 38.4 (36.0) | 23.9, 53.0 | 45.4 (39.0) | 28.5, 62.2 | .071 |
| Pleasurable (0-100) | 33.1 (29.6) | 20.3, 45.9 | 54.2 (38.4) | 38.7, 69.7 | 53.0 (35.3) | 20.7, 49.3 | 40.7 (35.4) | 25.3, 56.0 | .022 |
| Satisfying (0-100) | 43.7 (32.2) | 29.7, 57.6 | 54.5 (39.8) | 38.4, 70.6 | 51.5 (40.4) | 35.2, 67.9 | 46.2 (38.9) | 29.3, 63.0 | .621 |
| Interest in future use (0-100) | 33.4 (35.7) | 18.0, 48.9 | 48.3 (41.4) | 31.6, 65.0 | 35.3 (39.8) | 19.3, 51.4 | 46.7 (40.1) | 29.3, 64.0 | .055 |
| Willingness for future use (0-100) | 37.2 (35.6) | 21.8, 52.6 | 47.8 (39.0) | 32.1, 63.6 | 38.9 (38.4) | 23.4, 54.4 | 46.6 (40.5) | 29.1, 64.1 | .172 |
| *mCEQ subscales* | | | | | | | | | |
| Satisfaction (1-21) | 9.0 (5.0) | 6.9, 11.2 | 11.3 (5.7) | 9.0, 13.6 | 9.8 (6.2) | 7.4, 12.3 | 10.1 (5.9) | 7.5, 12.7 | .176 |
| Respiratory sensation (1-7) | 2.9 (2.0) | 2.0, 3.7 | 3.3 (2.2) | 2.5, 4.2 | 2.8 (2.0) | 2.0, 3.6 | 2.9 (2.1) | 2.0, 3.8 | .374 |
| *Behavioral intentions* | | | | | | | | | |
| Try flavor again (1-5) | 3.2 (1.1) | 2.7, 3.7 | 3.3 (1.3) | 2.8, 3.9 | 3.3 (1.5) | 2.7, 3.9 | 3.3 (1.5) | 2.7, 3.9 | .731 |
| Pay to use flavor (1-5) | 2.3 (1.4) | 1.7, 2.9 | 2.9 (1.3) | 2.4, 3.4 | 2.8 (1.6) | 2.2, 3.5 | 2.7 (1.6) | 2.0, 3.3 | .380 |
| Purchase for personal use (1-5) | 2.2 (1.3) | 1.6, 2.8 | 3.0 (1.4) | 2.5, 3.6 | 2.9 (1.5) | 2.3, 3.5 | 2.8 (1.4) | 2.2, 3.4 | .138 |
| Use flavor regularly (1-5) | 2.3 (1.4) | 1.7, 2.9 | 2.8 (1.3) | 2.3, 3.4 | 2.5 (1.5) | 1.9, 3.1 | 2.9 (1.5) | 2.2, 3.5 | .077 |
| *Craving and withdrawal* | | | | | | | | | |
| Craving change | -12.4 (14.7) | -18.7, -6.0 | -10.6 (14.0) | -16.3, -4.9 | -11.1 (14.0) | -16.8, -5.5 | -7.7 (12.9) | -13.2, -2.1 | .123 |
| Withdrawal change | -3.4 (4.3) | -5.3, -1.6 | -0.6 (3.0) | -1.8, 0.6 | -2.3 (4.8) | -4.3, -0.4 | -2.3 (3.5) | -3.9, -0.8 | .056 |
| *Notes.* IPI = interpuff interval. mCEQ = Modified Cigarette Evaluation Questionnaire. *Max puff volume values are log transformed to correct violations of the normality assumption. Periods describe the order in which participants were randomized to use the two e-liquid flavor. Periods were separated by a standard 90-minute washout period. | | | | | | | | | |
